# Carriage of vancomycin-resistant *Enterococcus faecium* in infants following an outbreak in the neonatal intensive care unit: time to clearance of carriage and use of molecular methods to detect colonization

**DOI:** 10.1017/ice.2021.524

**Published:** 2023-03

**Authors:** Vered Schechner, Jonathan Lellouche, Sarit Stepansky, Dror Mandel, Galia Grisaru-Soen, Liat Wullfhart, David Schwartz, Yehuda Carmeli

**Affiliations:** 1 Division of Epidemiology and Preventive Medicine, Tel Aviv Sourasky Medical Center, Israel; 2 Sackler Faculty of Medicine, Tel Aviv University, Tel Aviv, Israel; 3 National Institute for Antibiotic Resistance and Infection Control, Ministry of Health, Tel Aviv, Israel; 4 The Adelson School of Medicine, Ariel University, Ariel, Israel; 5 Department of Neonatology, Dana Dwek Children’s Hospital, Tel Aviv Sourasky Medical Center, Israel; 6 Pediatric Infectious Disease Unit, Dana Dwek Children’s Hospital, Tel Aviv Sourasky Medical Center, Israel

## Abstract

Among 46 infants colonized with vancomycin-resistant *Enterococcus faecium* during an outbreak in a neonatal intensive care unit, the estimated time until half had achieved clearance was 217 days. All 40 infants who completed follow-up cleared carriage by 1 year. No predictors of prolonged carriage (> 6 months) were identified.

Acquired resistance to vancomycin among *Enterococcus faecium* and *E. fecalis* is a public health concern. The WHO classified vancomycin-resistant *E. faecium* (VRE-fm) as a pathogen of high priority in its list of antibiotic-resistant bacteria for which research and development of new antibiotics are needed.^1^

VRE fecal colonization often precedes infection.^2^ Duration of fecal carriage among adult patients may last months and years, and may depend on antibiotic exposure and interaction with the healthcare system after hospital discharge.^3^ Reports of VRE outbreaks in neonatal intensive care units (NICU) are few,^4–7^ and data about the duration of carriage after NICU discharge are lacking.

In 2017, a monoclonal outbreak of VRE-fm harboring the *vanA* gene involving 49 NICU patients occurred at Tel Aviv Sourasky Medical Center (TASMC), Israel.^8^ In all cases, the infants had only VRE colonization, without clinical infection. To determine colonization status, stool samples were taken periodically after discharge to inform parents and end the need for contact isolation if infants continued interactions with the healthcare system. In this study, we aimed (1) to determine time to clearance of VRE-fm carriage after discharge from NICU, (2) to identify factors associated with prolonged carriage, and (3) to compare the time to clearance using molecular versus culture-based methods.

## Methods

### Setting, study design, and sample

The sample for this retrospective cohort study consisted of the 49 newborns in the NICU who were identified as colonized with VRE-fm between February 20, 2017, and April 11, 2017. After discharge, the infants underwent sequential stool tests to determine VRE clearance. For this study, follow-up ended on September 1, 2018.

### Data collection

Data on characteristics of the infants, the delivery, and the NICU hospitalization were collected from electronic medical records. Antimicrobial agents given during hospitalization in the NICU were divided into first-line (ampicillin and gentamicin) and second-line antibiotics (all others). Results of stool tests for VRE and any clinical cultures taken during the follow-up period were examined.

### Microbiological methods and testing protocol

VRE-fm was tested on fresh stool specimens. VRE-fm presence was determined after enrichment in brain heart infusion (BHI) broth by 2 different methods: standard microbiological culture using selective and chromogenic media (CHROMagar VRE, Mast Diagnostica GmbH, Reinfeld, Germany) and PCR detection of the *van*A and housekeeping *ddl*-fm genes (see Supplementary Material online).

A positive stool test for VRE-fm was defined as a positive culture for VRE-fm and/or positive PCR tests for both *van*A and *ddl*. A stool test was defined as negative if all 3 tests were negative or if the culture was negative and either *van*A or *ddl* was negative. Testing began no sooner than 3 months after the initial positive test. A negative test was confirmed by repeat testing 2 weeks later. A positive test was followed by repeat testing 2 months later.

### Outcome

The outcome of interest was time until VRE-fm clearance. Clearance was defined as negative stool tests for VRE-fm on two consecutive specimens at least 2 weeks apart. The date of the initial positive VRE-fm test was the starting point and the date of the first of the 2 negative tests was the end point. We also calculated time until VRE clearance based on culture results only.

### Statistical analysis

Median time to clearance was determined using a nonparametric maximum likelihood estimation (NPMLE) of survival. A paired *t* test was used to compare time to clearance based on culture versus culture plus PCR. The χ^2^ test and multivariable logistic regression were used to identify risk factors for prolonged VRE carriage (>6 months). Analyses were performed using Python version 3.7.4 software (Python, Wilmington, DE) and R Studio version 3.6.3 software (R Foundation for Statistical Computing, Vienna, Austria).

### Ethics

This study was approved by the TASMC Institutional Review Board.

## Results

### Patient characteristics

Among the 49 infants studied, 34 were preterm. Median length of stay was 24 days (IQR 12–45 days). Antibiotics were given to 80% of the infants received antibiotics. Patients are described in detail in Table S1 (online).

### Duration of VRE-fm colonization

**Fig. 1.**
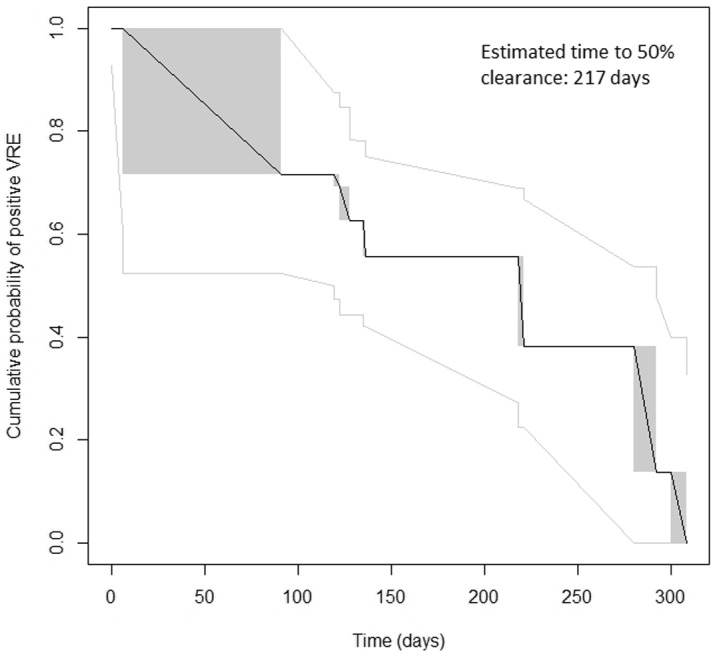
Nonparametric maximun likelihood (NPMLE) of survival for the duration of VRE-fm carriage, based on culture and PCR tests. The gray boxes indicate the intervals during which VRE clearance was not tested. The grey lines are confidence intervals.

In total, 46 patients performed stool tests for VRE clearance after discharge (range, 1–8 tests). Among them, 6 patients were lost to follow-up before clearance was achieved. Thus, 40 patients cleared VRE as defined by culture and PCR after a median of 4 tests. Time from initial positive test to clearance ranged between 91 and 361 days. The estimated time until 50% of patients achieved clearance was 217 days (Fig. 1).

Based on culture alone, 41 patients achieved clearance between 91 and 294 days after the initial positive test. The estimated time until half of patients achieved clearance was 113 days (Fig. S1). The difference in time to clearance using the two methods was significant (*P* < .0001).

After 1 negative test, the next test was also negative in 32 (80%) of 40 patients. A negative culture was followed by a positive culture for VRE-fm in only 1 case; the negative culture test was positive for *van*A and negative for *ddl.* During a follow-up period of 16–18 months, no patients had a VRE clinical infection detected by our hospital’s laboratory.

### Factors associated with prolonged carriage

Time to VRE-fm clearance was ≤6 months for 19 patients and >6 months for 26 patients. In bivariate analysis, mechanical ventilation, having a central line, receiving an oral iron supplement, and receiving second-line antibiotics protected against prolonged VRE-fm carriage (*P* < .10), but none remained significant in multivariable analysis (Table 1).

**Table 1.** Association Between Patient Characteristics and Duration of VRE-fm Carriage^a^

Variables	Carriage ≤6 mo (n=19), No. (%)	Carriage > 6 mo (n=26), No. (%)	Bivariate Analysis		Multivariate Analysis	
			OR (95% CI)	*P* Value	OR (95% CI)	*P* Value
**Baseline characteristics**						
Female	8 (42.1)	15 (57.7)	1.88 (0.57–6.21)	.302		
Gestational age, weeks						
Extremely and very preterm (<32)	6 (31.6)	7 (26.9)	0.73 (0.15–3.47)	.691		
Moderate to late preterm (32 0/7 to 36 6/7)	8 (42.1)	11 (42.3)	0.86 (0.20–3.64)	.821		
Term (≥37)	5 (26.3)	8 (30.8)	Reference			
Birth weight, g						
<1,500	7 (36.8)	7 (26.9)	0.70 (0.17–2.91)	.623		
1,500–2,500	5 (26.3)	9 (34.6)	1.26 (0.29–5.42)	.445		
>2,500	7 (36.8)	10 (38.5)	Ref			
Delivery by caesarean section	12 (63.2)	14 (53.8)	0.68 (0.20–2.28)	.532		
Rupture of membranes ≥12 hours	3/18 (16.7)	5/25 (20.0)	1.25 (0.26–6.07)	.782		
Maternal antibiotic exposure during delivery	16/18 (88.9)	19/25 (76.0)	0.40 (0.07–2.24)	.284		
Is part of twins	3/18 (16.7)	6/26 (23.1)	1.50 (0.32–7.00)	.604		
Having siblings	10/17 (58.8)	15/25 (60.0)	1.05 (0.30–3.68)	.939		
Any comorbidity^b^	13 (68.4)	17 (65.4)	0.87 (0.25–3.08)	.83		
**Hospitalization characteristics**						
Length of stay ≥30 days	12 (63.2)	10 (38.5)	0.37 (0.11–1.24)	.102		
Time from initial VRE positive test to discharge ≥14 d	4/18 (22.2)	8/25 (32.0)	1.65 (0.41–6.63)	.48		
Mechanical ventilation	9 (47.4)	4 (15.4)	0.20 (0.05–0.82)	.019	0.28 (0.06–1.37)	.116
Presence of CVC	12 (63.2)	9 (34.6)	0.31 (0.09–1.06)	.058	0.44 (0.08–2.54)	.364
NGT ≥10 d	10/18 (55.6)	12 (46.2)	0.69 (0.21–2.30)	.540		
TPN days > 0	11 (57.9)	9 (34.6)	0.39 (0.11–1.30)	.121		
Breast milk (≥50% of hospital days)	11 (57.9)	17/23 (73.9)	2.06 (0.56–7.58)	.273		
Receipt of PPI or H2RA	4/17 (23.5)	4 (15.4)	0.59 (0.13–2.77)	.502		
Erythropoietin therapy	4/18 (22.2)	6 (23.1)	1.05 (0.25–4.42)	.947		
Oral iron supplement	13/18 (72.2)	12 (46.2)	0.33 (0.09–1.20)	.086	0.94 (0.14–6.15)	.946
Antibiotic treatment (≥10 d of therapy)	7/18 (38.9)	6 (23.1)	0.47 (0.13–1.76)	.258		
First-line antibiotics	16 (84.2)	18 (69.2)	0.42 (0.10–1.87)	.248		
Second-line antibiotics	9 (47.4)	6 (23.1)	0.33 (0.09–1.20)	.088	0.76 (0.14–4.18)	.750
IV vancomycin	4 (21.1)	1 (3.8)	0.15 (0.02–1.47)	.070		

Note: CVC, central venous catheter; NGT, nasogastric tube; TPN, total parenteral nutrition; PPI, proton-pump inhibitor; H_2_RA, histamine-2 receptor antagonist.

a Denominators are listed if data were missing.

b Respiratory distress syndrome or broncho-pulmonary dysplasia or intraventricular hemorrhage or cardiovascular disease or retinopathy of prematurity or anemia of prematurity or neonatal jaundice or necrotizing enterocolitis.

## Discussion

To our knowledge, this is the first study to show the natural history of VRE carriage among NICU patients after discharge from the hospital. This information is important to inform concerned parents of colonized infants, and it may help clinicians and infection control teams. At 90 days, ∼30% of infant carriers cleared carriage. In 1 year, all 40 patients who completed follow-up had cleared carriage.

In another study that examined VRE carriage duration among adults, median time from discharge to first negative culture was 33 days, as compared to 113 days by culture in our study.^9^ The difference between the 2 studies may reflect differences in the start date (initial detection date versus discharge date) and in laboratory methods. It may also reflect differences between adults and infants in maturity of the microbiome, whose restoration is important to support clearance of carriage.

Our patients were not tested until at least 3 months after initial detection, so clearance might have occurred earlier in some patients. However, 31 of 46 were still carriers at their first follow-up test, which is similar to other reports of >50% positivity 3–6 months after initial VRE detection.^10^ Given these findings, we recommend testing colonized infants for VRE clearance beginning at 3 months after the first positive test.

Strengths of our study include a unique cohort of infants involved in a clonal VRE-fm outbreak, high compliance with follow-up testing, and a sensitive method for VRE detection. This study had several limitations. First, this was not a prospective study in which infants were retested at uniform intervals. Second, PCR tests may be falsely positive if the genetic resistance element was detected in nonenterococcal organisms or a nonviable *Enterococcus*. To minimize the risk of false positives, we defined PCR testing as positive if both *van*A and *ddl* genes were present. Third, we had no information on potential risk factors for prolonged carriage after discharge

In summary, we have shown that infants who acquired VRE-fm during an outbreak in the NICU cleared carriage by 1 year after initial diagnosis.

## Supplementary material

For supplementary material accompanying this paper visit https://doi.org/10.1017/ice.2021.524. click here to view supplementary material
