# Supplementary material for: Carriage of vancomycin-resistant Enterococcus faecium in infants following an outbreak in the neonatal intensive care unit: time to clearance of carriage and use of molecular methods to detect colonization
Source: Infect Control Hosp Epidemiol. 2022 Jan 31;44(3):497–500. doi: 10.1017/ice.2021.524 (PMC10015260; doi:10.1017/ice.2021.524)

Supplementary material for "Carriage of vancomycin-resistant *Enterococcus faecium* in infants following an outbreak in the neonatal intensive care unit: time to clearance of carriage and use of molecular methods to detect colonization"

1) Laboratory methods

2) Table S1

3) Figure S1

**Laboratory methods for VRE-fm detection**

*Molecular approach*

The molecular method was previously described by Depardieu et al.^19^ A multiplex PCR reaction was used to detect both *van*A and *ddl* target genes. *ddl* gene was chosen for the specific detection of *E. faecium* and *van*A gene to confirm the presence of a resistance gene to vancomycin. PCR products were sequenced using a Sanger method (HyLabs, Rehovot, Israel) and analyzed by BLAST. Three well-characterized strains from our collection were used as quality control strains: (1) *E. faecium*-*van*A positive, (2) *E. faecium*-*van*A negative and (3) *E. faecalis*-*van*B positive.

*Culture approach*

Each stool specimen was inoculated in 5mL Brain Heart Infusion (BHI) enrichment broth to increase sensitivity. Samples were incubated aerobically at 35-37°C for 18 hours before inoculation on CHROMagar VRE™ plate. An additional incubation of 18 hours was performed.

Suspected pink colonies were identified using a VITEK^®^ 2 system (bioMérieux, Marcy l'Etoile, France). Initial susceptibility including vancomycin was obtained by the VITEK^®^ 2 system. In the case of *E. faecium* identification and MIC VAN >4µg/mL, vancomycin reduced susceptibility or resistance was confirmed by disk diffusion [CLSI M02QGE]. Susceptibility was interpreted using CLSI guidelines [CLSI M100]. In the case of discrepancy between VITEK^®^ 2 and disk diffusion results, the susceptibility obtained by the gold standard method (i.e disk diffusion) was reported. *S. aureus* ATCC^®^ 259923 and *E. faecalis* ATCC^®^ 29212 were used as quality control strains.

Table S1. Baseline and hospitalization characteristics of the 49 study patients (Denominators are listed if data were missing).

| Baseline characteristics |  |
| --- | --- |
| Female, N (%) | 27 (55.1%) |
| Gestational age, median (IQR) | 35.4 (31.3, 37.7) |
| Extremely preterm (<28), N (%) | 7 (14.3%) |
| Very preterm (28 0/7 – 31 6/7), N (%) | 6 (12.2%) |
| Moderate to late preterm (32 0/7 – 36 6/7), N (%) | 21 (42.9%) |
| Term (≥37), N (%) | 15 (30.6%) |
| Birth weight, mean (± SD) | 2166.4 (±1044.5) |
| <1500g, N (%) | 14 (28.6%) |
| 1500-2500g, N (%) | 16 (32.7%) |
| >2500g, N (%) | 19 (38.8%) |
| Delivery by Caesarean section, N (%) | 28 (57.1%) |
| Rupture of membranes ≥12 hours, N (%) | 8/46 (17.4%) |
| Maternal antibiotic exposure during delivery, N (%) | 38/47 (80.9%) |
| Is part of twins, N (%) | 9/48 (18.8%) |
| Having siblings, N (%) | 26/46 (56.5%) |
| Co-morbidities, N (%) |  |
| Respiratory distress syndrome | 13/46 (28.3%) |
| Broncho-pulmonary dysplasia | 6/47 (12.8%) |
| Intraventricular hemorrhage | 5/47 (10.6%) |
| Cardiovascular disease | 20/49 (40.8%) |
| Retinopathy of prematurity | 4/47 (8.5%) |
| Anemia of prematurity | 16/48 (33.3%) |
| Jaundice | 16/47 (34.0%) |
| Necrotizing enterocolitis | 4/47 (8.5%) |
| Hospitalization characteristics |  |
| Length of stay, days, median (IQR) | 24 (12.0, 45.0) |
| Length of stay ≥30 days, N (%) | 22 (44.9%) |
| Mechanical ventilation, N (%) | 13 (26.5%) |
| Mechanical ventilation days, mean (± SD)* | 9.2 (±12.4) |
| Presence of CVC, N (%) | 22 (44.9%) |
| Central line days, mean (± SD)* | 26 (±17.2) |
| NGT ≥10 days, N (%) | 22/48 (45.8%) |
| NGT days, mean (± SD)* | 31.3 (±39.2) |
| TPN, N (%) | 21 (42.9%) |
| TPN days, mean (± SD) * | 21.8 (±16.2) |
| Breast milk (on ≥50% of hospital days), N (%) | 29/46 (63.0%) |
| Receipt of PPI or H_2_RA, N (%) | 8/47 (17.0%) |
| Erythropoietin therapy, N (%) | 10/48 (20.8%) |
| Oral iron supplement, N (%) | 27/48 (56.2%) |
| Antibiotic treatment, days, mean (± SD) | 11.7 (±19.2) |
| First-line antibiotics, N (%) | 36 (73.5%) |
| Second-line antibiotics, N (%) | 16 (32.7%0 |

Note: CVC, central venous catheter; NGT, naso-gastric tube; TPN, total parenteral nutrition; PPI, proton-pump inhibitor; H_2_RA, histamine-2 receptor antagonist; first-line antibiotics are ampicillin and gentamicin, all others are second line.

* Only positive cases included

**Figure S1.** Nonparametric Maximum Likelihood (NPMLE) of Survival for the duration of VRE-fm carriage, based on culture results


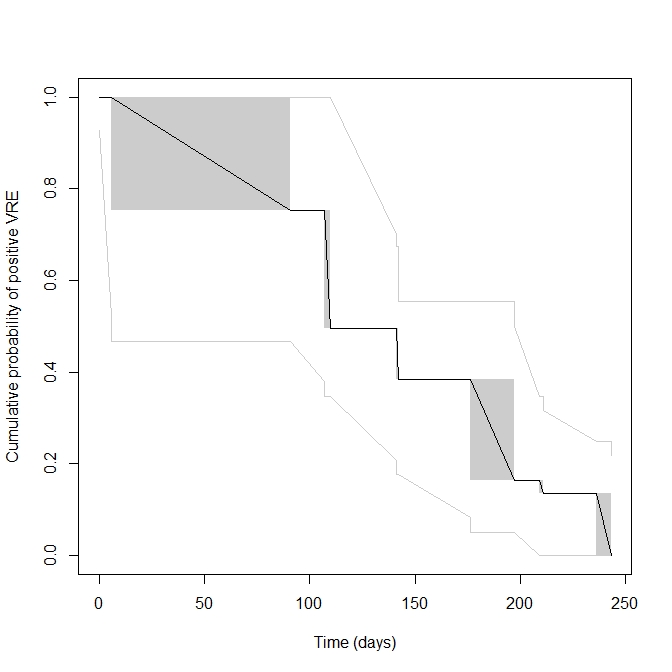

Supplement: Supplementary file 1 [file S0899823X21005249sup001.docx]
